# Genome-wide association study identifies new loci for albuminuria in the Japanese population

**DOI:** 10.1007/s10157-020-01884-x

**Published:** 2020-07-20

**Authors:** Hiroshi Okuda, Koji Okamoto, Michiaki Abe, Kota Ishizawa, Satoshi Makino, Osamu Tanabe, Junichi Sugawara, Atsushi Hozawa, Kozo Tanno, Makoto Sasaki, Gen Tamiya, Masayuki Yamamoto, Sadayoshi Ito, Tadashi Ishii

**Affiliations:** 1 Department of Education and Support for Regional Medicine, Tohoku University Hospital, 1-1 Seiryo-machi, Aoba-ku, Sendai, Miyagi 980-8574 Japan; 2 Tohoku Medical Megabank Organization, Tohoku University, 2-1 Seiryo-machi, Aoba-ku, Sendai, Miyagi 980-8573 Japan; 3 Department of Nephrology, Endocrinology and Vascular Medicine, Graduate School of Medicine, Tohoku University, 1-1 Seiryo-machi, Aoba-ku, Sendai, Miyagi 980-8574 Japan; 4 Radiation Effects Research Foundation, 5-2 Hijiyama Park, Minami-ku, Hiroshima, Hiroshima, 732-0815 Japan; 5 Iwate Tohoku Medical Megabank Organization, Iwate Medical University, 1-1-1 Idaidori, Yahaba-cho, Shiwa-gun, Iwate, 028-3694 Japan; 6 RIKEN Center for Advanced Intelligence Project Nihonbashi, 1-chome Mitsui Bldg. 15F, 1-4-1 Nihonbashi, Chuo-ku, Tokyo, 103-0027 Japan

**Keywords:** QTL, GWAS, Albuminuria, Genetics, TSHR, Cohort study

## Abstract

**Background:**

Urinary albumin excretion (UAE) is a risk factor for cardiovascular diseases, metabolic syndrome, chronic kidney disease, etc. Only a few genome-wide association studies (GWAS) for UAE have been conducted in the European population, but not in the Asian population. Here we conducted GWAS and identified several candidate genes harboring single nucleotide polymorphisms (SNPs) responsible for UAE in the Japanese population.

**Methods:**

We conducted GWAS for UAE in 7805 individuals of Asian ancestry from health-survey data collected by Tohoku Medical Megabank Organization (ToMMo) and Iwate Tohoku Medical Megabank Organization (IMM). The SNP genotype data were obtained with a SNP microarray. After imputation using a haplotype panel consisting of 2000 genome sequencing, 4,962,728 SNP markers were used for the GWAS.

**Results:**

Eighteen SNPs at 14 loci (*GRM7*, *EXOC1*/*NMU*, *LPA*, *STEAP1B*/*RAPGEF5*, *SEMA3D*, *PRKAG2*, *TRIQK*, *SERTM1*, *TPT1-AS1*, *OR5AU1*, *TSHR*, *FMN1*/*RYR3*, *COPRS,* and *BRD1*) were associated with UAE in the Japanese individuals. A locus with particularly strong associations was observed on *TSHR*, chromosome 14 [rs116622332 (*p* = 3.99 × 10^−10^)].

**Conclusion:**

In this study, we successfully identified UAE-associated variant loci in the Japanese population. Further study is required to confirm this association.

**Electronic supplementary material:**

The online version of this article (10.1007/s10157-020-01884-x) contains supplementary material, which is available to authorized users.

## Introduction

Chronic kidney disease (CKD) is one of the most severe global public health problems [1]. The proportion of patients with end-stage renal disease is growing, and thus, the resultant cost poses a big problem in health economics [2]. It is important to diagnose renal failure in the early stages to prevent disease progression. However, it is very difficult to do so as the typical symptoms of renal failure rarely emerge in the earlier stages. The risks of mortality, myocardial infarction, and progression to kidney failure associated with a particular value of estimated glomerular filtration rate (eGFR) are increased independently in patients with moderate to severe urinary albumin excretion (UAE) [3]. Apart from CKD, UAE is known biomarker of cardiovascular diseases, diabetes mellitus, obesity, hypertension, and all-cause mortality [4–8]. Even albuminuria of less than 30 mg/gCr (lower than microalbuminuria) is known as a marker of these diseases [7, 8].

While a few genome-wide association studies (GWAS) on UAE have been conducted in individuals with type 2 diabetes mellitus [9] and type 1 diabetes mellitus [10] in European ancestral cohorts [11–13], there is no such GWAS conducted in the Asian population. Here we have conducted GWAS using health-survey data collected in the Tohoku Medical Megabank Organization (ToMMo) and Iwate Tohoku Medical Megabank Organization (IMM) to identify the several candidate genes harboring single nucleotide polymorphisms (SNPs) responsible for UAE in the Japanese population.

## Material and methods

### Study subjects

This research was conducted as a part of the residential cohort study of Tohoku Medical Megabank (TMM), a joint organization of the Tohoku University and the Iwate Medical University in Japan, established in 2011 after the Great East Japan Earthquake for creating an advanced medical system.

Over 80,000 almost healthy adult individuals living in the Miyagi and Iwate Prefectures along the Pacific coast of the Tohoku district of northern Japan were recruited from May 2013 to March 2016 for the TMM Project. The participants were of 20–75 years old and completed questionnaires covering a wide range of topics including socio-demographic factors, lifestyle habits, and medical history. Blood and urine tests were performed at baseline survey. The participants living in the Miyagi Prefecture and Iwate Prefecture were recruited by Tohoku University and Iwate Medical University, respectively [14, 15]. We obtained approval from the relevant ethics committees of both the facilities. We obtained written informed consent from each participant when they were enrolled in the TMM cohort study. This study was conducted according to the principals of the Declaration of Helsinki.

From the 10,000 individuals whose data were collected up to 2013, we were able to obtain data of 9,966 individuals after excluding 34 people who withdrew their consent after collection. The data were released as dbToMMo 1.1. Among these 9,966 individuals, 4974 were from the Miyagi prefecture and 4992 were from the Iwate prefecture. Thus, both represented a roughly equal proportion.

### Sample quality control

Genotyping was performed for 964,193 SNP markers using Illumina's Human Omni Express Exome- 8 version 1.2 BeadChips. Upon conducting quality control of the samples based on the genotyping data, some people were excluded owing to data loss (*n* = 1), genotype defect (low call rate: call rate < 0.98, *n* = 5), or close relationship pairs (identity-by-descent estimates, PI_HAT > 3/32, *n* = 2155) [16, 17]. Finally, the data sampled from 7805 individuals passed quality control.

### Marker quality control

As quality control of the genotyped marker, SNPs with low call rates (< 0.95), and low *p* values in the Hardy Weinberg equilibrium (HWE) test (*p* value < 1.0 × 10^−4^), low minor allele frequencies (MAF < 0.01), and low-quality markers among the duplication markers were filtered out. As a result, 595,171 SNPs remained for the downstream analysis.

### Genotype imputation

Genotype imputation was performed using SHAPEIT v2.r837 [18] and IMPUTE2 v2.2.2 [19] software packages with TMM 2KJPN high-quality haplotype reference panel based on the 2049 drafts of the whole genome sequencing and was implemented in the TMM [20]. After genotype imputation, we adjusted the imputation quality (INFO scores) and MAF. The variants with low imputation quality (INFO scores < 0.5) and low minor allele frequency (MAF < 0.03) variants were excluded. Ultimately, 4,962,728 variants were retained for the GWAS.

### Phenotype

Phenotype information was obtained from the questionnaires covering age, sex, physical measurement including body mass index (BMI), and systolic blood pressure (SBP). For standardizing the blood pressure estimation, we did not use the antihypertensive medication history because systolic blood pressure strongly influences the glomerular pressure and UAE [21].

Urinary Na (UNa), urinary K (UK), urinary creatinine (UCr), urinary albumin (Ualb), serum creatinine (sCre), serum cystatin C (sCysC), hemoglobin A1c (HbA1c), and eGFR were estimated at baseline. We selected eGFR calculated by serum cystatin C (eGFRcys) for the evaluation of renal functional instead of eGFR calculated by serum creatinine (eGFRcre) because serum cystatin C was a better marker of early-stage CKD than serum creatinine [22]. eGFRcys was calculated by the Japanese equation for eGFR from serum cystatin C as follows [23, 24];

$${\text{eGFRcys mL/min/1}}{\text{.73 m}}^{2} = 104 \times {\text{SCysC}}^{{ - 1.019}} {\mkern 1mu} \times {\mkern 1mu} 0.996^{{{\text{age}}}} {\mkern 1mu} \times {\mkern 1mu} 0.929(if \,female)-8$$

### Statistical analyses

After performing a standard linear regression analysis of UAE (PLINK version 1.9 software package) for each SNP, we performed GWAS for UAE. We used UAE corrected by creatinine (continuous variable) as a response variable. The analysis was adjusted for the relevant covariates including age, sex, BMI [25], SBP [26], UNa [27], UK [27], HbA1c [26], eGFRcys [28], and the top significant 26 principal components of the genotypes. These are reported confounding factors for albuminuria.

We constructed a Manhattan plot and Quantile–quantile plot (Q-Q plot) to visually evaluate the analysis result. We used the statistical software R with “qqman” package. We constructed Regional plot to evaluate the linkage disequilibrium (LD) structure around the SNPs with Locus Zoom [URL; https://locuszoom.org]. We evaluated the result of the expression Quantitative Trait Loci (eQTL) analysis for some genome-wide significant SNPs by the Genotype Tissue Expression Project (GTEx) portal [29].

## Results

### Basic characteristics of the study subjects

The genotype data from 7805 individuals passed quality control and were used for the analysis. The detailed characteristics of the analyzed data are shown in Table 1. There were 60.7% patients of CKD stage 1, 36.6% patients of CDK stage 2, and 2.71% patients of CKD stage ≥ 3. In this setting, cystatin C seemed to be more appropriate for kidney function marker. The mean age of the patients was 61.8 ± 11.2 years, and 34.8% of the patients were male. The average systolic blood pressure of these patients was 127 ± 17.8 mm Hg and the median of UAE was 7.4 mg/gCr [interquartile range (IQR) 8.8]. About one-fourth (24.5%) of the patients were hypertensive [defined as systolic blood pressure > 140 mmHg or diastolic blood pressure > 90 mmHg in accordance with The Japanese Society of Hypertension Guidelines for the Management of Hypertension (JSH 2014)] [30]. In this study, the individuals taking antihypertensive medications were also diagnosed as hypertensive.

**Table 1:** Demographic characteristics of the study population

Characteristics	Total	Ualb –	Ualb +
	*n* = 7805	*n* = 6970	*n* = 827
Age, years	60.8 ± 11.2	60.4 ± 11.5	64.8 ± 8.64
Sex, male (%)	2716 (34.8)	2349 (33.7)	360 (43.5)
BMI	23.5 ± 3.6	23.4 ± 3.52	24.6 ± 3.76
SBP, mmHg	127 ± 17.8	126 ± 17.2	137 ± 19.9
DBP, mmHg	75.4 ± 10.8	74.9 ± 10.6	79.7 ± 11.9
HTN_treat (%)	211 (2.7)	275 (2.53)	31 (3.79)
HTN_diag (%)	1912 (24.5)	1540 (22.1)	369 (44.6)
Ualb/UCr, mg/gCr (IQR)	7.4 (8.8)*	6.7 (6.2)*	64.7 (99)*
< 30 mg/gCr (%)	6970 (89.3)		
≧ 30 mg/gCr (%)	827 (10.6)		
UNa, g/l	3.02 ± 1.34	3.05 ± 1.28	2.91 ± 1.23
UK, g/l	1.63 ± 1.09	1.66 ± 1.04	1.50 ± 0.91
HbA1c(NGSP), %	5.56 ± 0.59	5.52 ± 0.54	5.81 ± 0.85
sCre, mg/dl	0.69 ± 0.24	0.68 ± 0.15	0.75 ± 0.43
sCysC, mg/l	0.77 ± 0.19	0.76 ± 0.15	0.85 ± 0.15
eGFRcre, ml/min/1.73 m^2^	78.1 ± 15.6	78.4 ± 0.15	74.8 ± 0.15
eGFRcys, ml/min/1.73 m^2^	97.4 ± 21.9	98.4 ± 0.15	89.5 ± 0.15
CKD stage 1 (%)	4737 (60.7)	4346 (62.4)	390 (47.2)
CKD stage 2 (%)	2857 (36.6)	2499 (35.9)	351 (42.4)
CKD stage 3a (%)	174 (2.23)	112 (1.60)	63 (7.58)
CKD stage 3b (%)	27 (0.35)	12 (0.17)	15 (1.83)
CKD stage 4 (%)	8 (0.10)	1 (0.01)	7 (0.86)
CKD stage 5 (%)	2 (0.03)	1 (0.01)	1 (0.12)

*SBP* systolic blood pressure, *DBP* diastolic blood pressure, *HTN_treat* the person treated as hypertension from questionnaire, *HTN_diag* the persons diagnosed based on The Japanese Society of Hypertension Guidelines for the Management of Hypertension (JSH 2014), *Ualb/UCr* urinary albumin excretion corrected by urinary creatinine, *UNa* urinary sodium, *UK* urinary potassium, *HbA1c(NGSP)* hemoglobin A1c valued as National Glycohemoglobin Standardization Program, *sCre* serum creatinine, *sCysC* serum cystatin C, *eGFRcre* estimated glomerular filtration rate calculated by serum creatinine, *eGFRcys* estimated glomerular filtration rate calculated by serum cystatin C, *CKD stage 1* eGFRcys ≥ 90, *CKD stage 2 * ≤ 60 eGFRcys < 90, *CKD stage 3a* 45 ≤ eGFRcys < 60, *CKD stage 3b* 30 ≤ eGFRcys < 45, *CKD stage 4* 15 ≤ eGFRcys < 30, *CKD stage 5* eGFRcys < 15, *Ualb − *the group without microalbuminuria nor overt albuminuria, *Ualb +* the group of microalbuminuria or overt albuminuria, *IQR* interquartile range

*Median value

About 10% of patients had UAE of > 30 mg/gCr, and hence most of the patients had microalbuminuria. The mean age of the UAE positive patients was 64.4 ± 8.64 years and 43.6% of these patients were males. The mean systolic blood pressure of these patients was 137 ± 19.9 mmHg and their average eGFRcys was 89.5 ± 23.7 ml/min/1.73 m^2^. The mean age of the UAE negative patients was 60.4 ± 11.5 years and 33.7% of these patients were male. The mean systolic blood pressure of these patients was 126 ± 17.2 mmHg and their average eGFRcys was 98.4 ± 21.3 ml/min/1.73 m^2^.

When we evaluate correlation between each covariant and urinary albumin excretion, there is no significant correlation (Table S1). When we evaluate correlation between each covariant and other covariant, there are weak correlations between age and eGFRcys (correlation factor 0.56), SBP and BMI (correlation factor 0.48), UNa and UK (correlation factor 0.44) (Table S2).

### Genome-wide association study for UAE in the Japanese populations

**Fig. 1:**
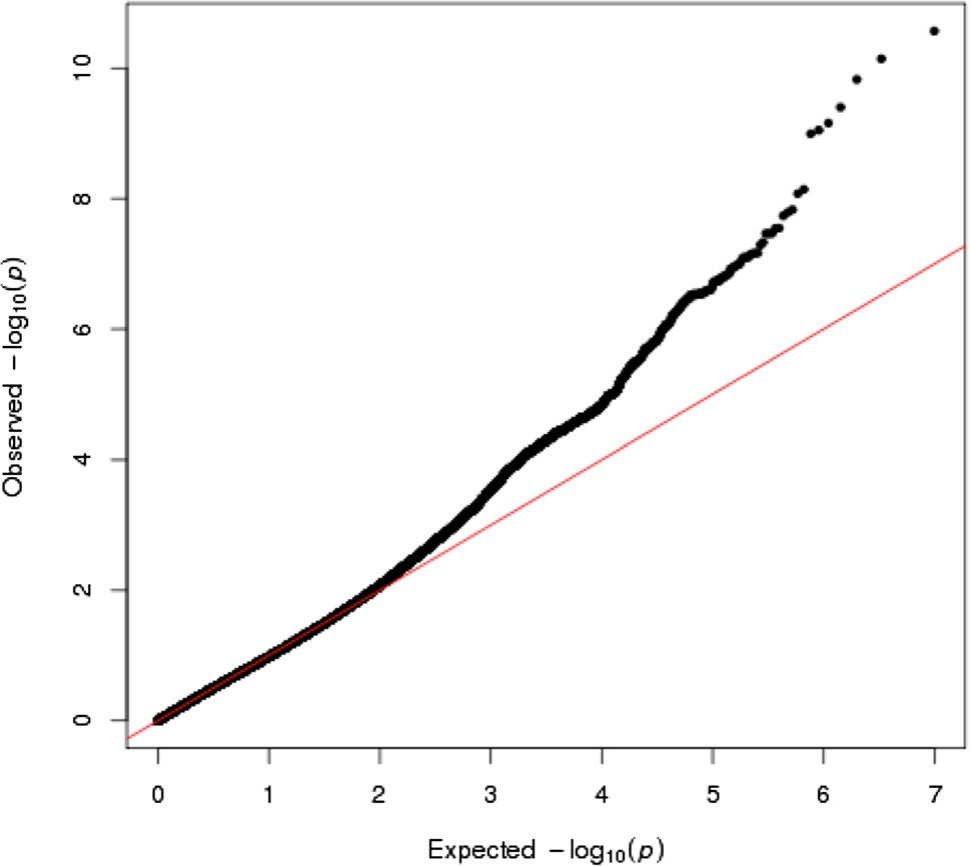
Q-Q plots of GWAS about UAE in the TMM cohort study. The negative logarithm of the observed (*y*-axis) and the expected (*x*-axis) *p*-value was plotted for each SNP (dot), and the red line (*y* = *x*) indicates the null hypothesis of no true association. The regression genomic inflation factor (λ score) is 0.987 (SE 6.07 × 10^−6^) to adequately control the population stratification

**Fig. 2:**
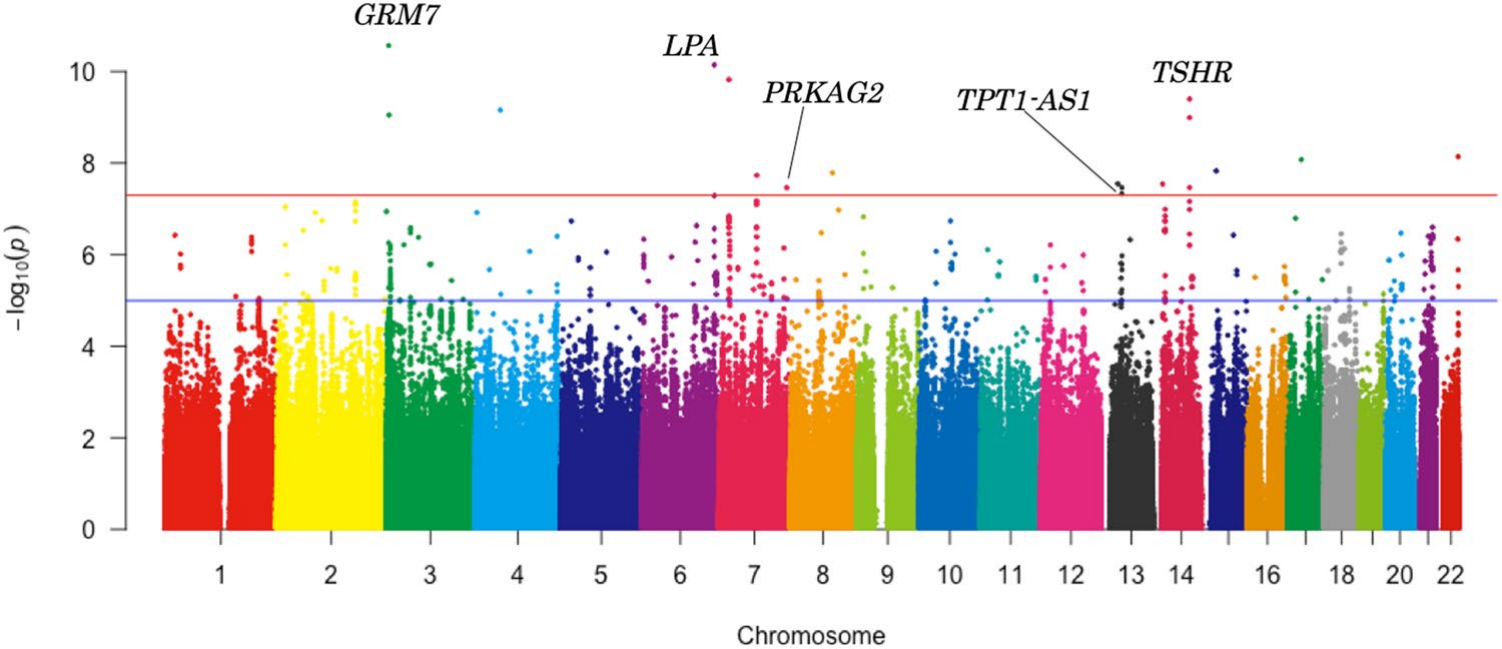
Manhattan plot of the GWAS for UAE in the TMM cohort study. The *X*-axis represents the chromosomal positions and the *Y*-axis represents the – log10 *p*-values. The red horizontal line indicates the genome-wide significance threshold of *p* = 5 × 10^−8^ and the blue horizontal line indicates the genome-wide suggestive threshold of *p* = 5 × 10^−5^. The name of the genes where the SNPs were located is typed in Manhattan plot

We performed GWAS in 7805 individuals. After genotyping, 595,171 SNPs passed the quality controls and were used for the following imputation analysis. For the imputation analysis, a haplotype reference panel based on the 2049 drafts of the whole genome sequencing was used. Finally, 4,962,728 variants were used for the GWAS. The Q-Q plot is shown in Fig. 1. The genomic inflation factor (λ) showed 0.987 suggesting that the population substructure should not have any substantial effects on the association analysis [14]. Under these conditions, we obtained 18 genome-wide significant SNPs (Table 2). We constructed a Manhattan plot of this GWAS as shown in Fig. 2.

**Table 2:** UAE associated SNPs reaching a genome-wide significance

Chr	SNP	Position	Gene(s)	ref	alt	EA	EAF	BETA	SE	INFO	*p* value	AR^2^
3	rs143146694	6,263,450	*GRM7*	G	T	T	0.035	37.81	5.667	0.931	2.69 × 10^–11^	0.034
3	rs74971332	7,058,507	***GRM7***	C	G	G	0.055	26.43	4.307	0.97	8.91 × 10^–10^	0.034
4	rs75938525	56,659,946	*EXOC1/NMU*	G	C	C	0.048	31.06	5.029	0.956	6.93 × 10^–10^	0.034
6	rs146871152	160,984,637	***LPA***	C	T	T	0.034	37.28	5.713	0.97	7.16 × 10^–11^	0.037
7	rs146418897	22,440,870	*STEAP1B/RAPGEF5*	T	C	C	0.033	36.85	5.743	0.941	1.49 × 10^–10^	0.034
7	rs140221313	84,600,098	*SEMA3D*	G	A	A	0.038	31.17	5.534	0.934	1.84 × 10^–8^	0.034
7	rs118160950	151,277,450	***PRKAG2***	C	T	T	0.036	30.48	5.517	0.975	3.43 × 10^–8^	0.036
8	rs141491217	94,068,096	*TRIQK*	A	G	G	0.039	33.01	5.84	0.932	1.63 × 10^–8^	0.035
13	rs79163227	37,208,221	*SERTM1*	A	G	G	0.056	25.92	4.664	0.941	2.84 × 10^–8^	0.035
13	rs142317900	45,963,584	***TPT1-AS1***	G	A	A	0.033	31.13	5.693	0.98	4.68 × 10^–8^	0.037
13	rs151183316	46,021,543	*TPT1-AS1*	C	T	T	0.035	30.6	5.54	0.981	3.43 × 10^–8^	0.035
14	chr14:21617499_TCTCA_T	21,617,499	*OR5AU1*	TCTCA	T	T	0.052	28.48	5.126	0.871	2.87 × 10^–8^	0.034
14	rs116622332	81,506,821	***TSHR***	T	C	C	0.046	30.67	4.897	0.98	3.99 × 10^–10^	0.036
14	rs199612558	81,508,922	***TSHR***	T	TA	TA	0.046	29.57	4.835	0.983	1.00 × 10^–9^	0.037
14	rs17111387	81,515,680	***TSHR***	C	T	T	0.068	22.24	4.025	0.996	3.42 × 10^–8^	0.037
15	rs140272046	33,494,078	*FMN1/RYR3*	G	A	A	0.042	33.38	5.886	0.886	1.47 × 10^–8^	0.034
17	rs148283070	30,129,004	*COPRS*	A	G	G	0.034	34.15	5.922	0.972	8.42 × 10^–9^	0.036
22	chr22:49949123_GA_G	49,949,123	*BRD1*	GA	G	G	0.052	27.83	4.805	0.945	7.22 × 10^–9^	0.034

*Chr* Chromosome, *SNP* single-nucleotide polymorphism, *Position* Chromosome position (GRCh37/hg19), *Gene* The name of Gene where the SNP is located, *ref* reference allele, *alt* alternative allele, *EA* effective allele, *EAF* effective allele frequency, *BETA* regression coefficient, *SE* Standard error of regression coefficient, *INFO* INFO score, *AR*^*2*^ Adjusted coefficient of determination. Bold type signifies that the SNPs were located on the gene. Normal type signifies that the SNPs were around the gene

**Fig. 3:**
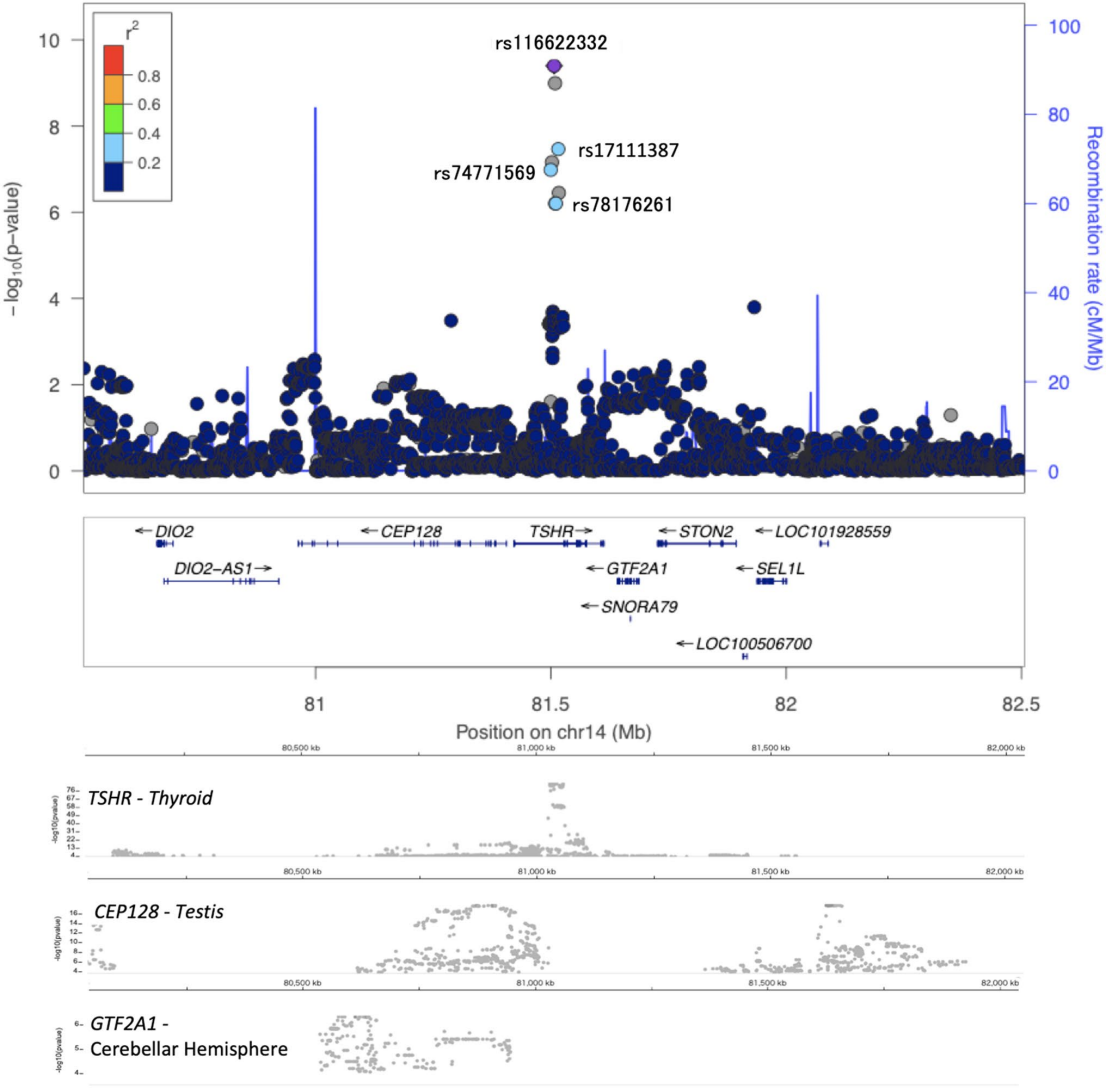
Association signals around the significant loci in *TSHR* locus. The upper panel is association signals around significant loci. The *X*-axis represents chromosomal positions (GRC37/hg19) and the *Y*-axis represents − log10 *p* -values. The lead variant is shown in purple. Colors represent the degree of LD (*r*^*2*^) between each variant and the lead variant. The LD (*r*^*2*^) was calculated based on the combined dataset of TMM subjects. The lower panels represent the Single-tissue eQTL analyses, where the target was mostly expressed. The data were from GTEx (V8). The *X*-axis is represents chromosomal positions (GRC38/hg38) and the *Y*-axis represents − log10 eQTL *p* -values. The *X*-axis between the upper and the lower panel is adjusted by calculating with hgLiftOver [https://genome.ucsc.edu/cgi-bin/hgLiftOver].

With respect to the SNPs meeting the significance level, we constructed a regional plot to visually examine LD with the surrounding SNPs (around 1 Mbp) and identified the gene in or around which the SNPs were located. We attached the result of eQTL analyses for genome-wide significant SNPs by GTEx (Fig. 3, Fig S1). Three SNPs were located in *TSHR* on chromosome 14q31 [rs116622332 (*p* = 3.99 × 10^−9^), rs199612558 (*p* = 1.00 × 10^−9^), and rs17111387 (*p* = 3.42 × 10^−8^)] (Fig. 3). Two SNPs were located in *GRM7* on chromosome 3p26.1 [rs143146694 (*p* = 2.69 × 10^−11^, and rs74971332 (*p* = 8.91 × 10^−10^) (Fig S1(a)). One SNP was located in *LPA* on chromosome 6q25.3 [rs146871152 (*p* = 7.16 × 10^–11^)] (Fig S1(b)). One SNP was located in *PRKAG2 *on chromosome 7q36.1 [rs118160950 (*p* = 3.43 × 10^−8^)] (Fig S1(c)). Two SNPs were located in *TPT1-AS1* on chromosome 13q14.13 [rs142317900 (*p* = 4.68 × 10^−8^) and rs151183316 (*p* = 3.43 × 10^−8^)] (Fig S1(d)). One SNP was located in *EXOC1/NMU* on chromosome 4q12 [rs75938525 (*p* = 6.93 × 10^−10^)] (Fig S1(e)). One SNP was located in *STEAP1B/RAPGEF5* on chromosome 7p15.3 [rs146418897 (*p* = 1.49 × 10^−10^)] (Fig S1(f)). One SNP was located in *SEMA3D* on chromosome 7q21.11 [rs140221313 (*p* = 1.84 × 10^−8^)] (Fig S1(g)). One SNP was located in *TRIQK* on chromosome 8q22.1 [rs141491217 (*p* = 1.63 × 10^−8^)] (Fig S1(h)). One SNP was located in *SERTM1* on chromosome 13q13.3 [rs79163227 (*p* = 2.84 × 10^−8^)] (Fig S1(i)). One SNP was located in *OR5AU1* on chromosome 14q11.2 [chr14:21617499_TCTCA_T (*p* = 2.87 × 10^−8^)] (Fig. 3j). One SNP was located in *FMN1/RYR3* on chromosome 15q13.3 [rs140272046 (*p* = 1.47 × 10^−8^)] (Fig S1(k)). One SNP was located in *COPRS* on chromosome 17q11.2 [rs148283070 (*p* = 8.42 × 10^−9^)] (Fig S1(l)). One SNP was located in *BRD1* on chromosome 22q13.33 [chr22:49949123_GA_G (*p* = 7.22 × 10^−9^)] (Fig S3(m)).

## Discussion

We performed GWAS for UAE in the Japanese general population and identified 18 SNPs, of which, 17 were not reported in any previous report. rs118160950 was already reported as a SNP related to UAE by GWAS performed in the European ancestry [31]. In the previous reports of GWAS for UAE, the study subjects were not a general cohort but consisted of diabetes patients, heart failure patients, or pregnant women with hypertension. In addition, the study subjects were mainly European or African American but not Asians. Our GWAS has profound significance among the Japanese general population.

In previous studies, rs10795433 [9] and rs1801239 [13] were reported as the significant SNPs associated with UAE. They are located on the *CUBN* gene locus*. CUBN* encodes cubilin protein acting as a receptor for vitamin B12-intrinsic factor complexes. It was hypothesized that the SNPs found in *CUBN* on chromosome 10 were significantly (*p* = 1.0 × 10^−11^) involved in UAE. However, these loci were not found to be significantly associated in our study. The reasons seem to be related to the difference in the studied population, because the reported significant covariates were not replicated in our population. We showed evidence of genetic differences between the Europeans and the East Asians. The other possibility is that these SNPs on *CUBN* are associated with UAE only in the diseased condition. There may be big differences in the mechanism between pathophysiological albuminuria and physiological albuminuria.

When we evaluated the functional class of 18 SNPs, 6 SNPs (rs74971332, rs146871152, rs118160950, rs116622332, rs199612558, and rs17111387) were intronic, 11 SNPs (rs143146694, rs75938525, rs146418897, rs140221313, rs141491217, rs79163227, rs151183316, chr14:21617499_TCTCA_T, rs140272046, rs148283070, and chr22:49949123_GA_G) were intergenic, and 1 SNP (rs142317900) was on the non-coding exons. No SNP was located on the coding exons. These candidates may possibly affect the factors regulating the transcription of the genes encoding the proteins involved in UAE.

In our study, the SNPs located in *TSHR* showed strong peaks. *TSHR* encodes TSHR (thyroid stimulating hormone receptor), and the TSH receptor is a member of the G protein-coupled receptor superfamily of integral membrane proteins which is coupled to the Gs protein [32, 33]. TSHR expresses mainly on the surface of the thyroid follicular cells and contributes to thyroid hormone secretion. Several pathways are proposed for kidney injury and proteinuria mediated by thyroid dysfunction [34, 35]. In hyperthyroidism, intra-glomerular hypertension, consequent hyperfiltration, increased production of free radicals, and increased renin–angiotensin–aldosterone system are risk factors for albuminuria. In hypothyroidism, GFR and tubular transport capacity are reduced. Hypothyroidism also results in increased glomerular capillary permeability to proteins directly causing proteinuria [36]. The other possibility is that TSHR could have direct effects on albumin re-uptake on the tubular cells of the kidney. TSHR is also expressed in other tissue, for e.g. adipose tissues and fibroblasts. It is known that a small amount of TSHR exists in the kidneys and mainly in the tubules [37–39]. We evaluated the expression of TSHR by immunohistochemistry in the renal biopsy specimens. We found a weak expression of TSHR in the kidney mainly in the tubules. A significant association between the staining level of TSHR and proteinuria was not detected (data not shown) and further study should be required to prove this concept.

*PRKAG2* coding 5′-AMP-activated protein kinase subunit gamma-2. AMP-activated protein kinase (AMPK) is a heterotrimeric protein composed of a catalytic alpha subunit, a noncatalytic beta subunit, and a noncatalytic regulatory gamma subunit [40]. AMPK is an important energy-sensing enzyme that monitors the cellular energy status and functions by inactivating the key enzymes involved in regulating the de novo biosynthesis of fatty acids and cholesterol. Mutations in this gene have been associated with WPW (Wolff-Parkinson-White) syndrome [41, 42], familial hypertrophic cardiomyopathy [43, 44], and enlarged kidneys [45]. Studies in transgenic mice indicate that these mutations cause glycogen storage disease of the heart [46]. Several other hereditary glycogen storage diseases present with renal pathologies, such as renal tubular dysfunction [47]. *PRKAG2* did not indicate any stronger significance in our GWAS. However, we may consider that renal tubular dysfunction induced by glycol storage can affect the UAE.

We used the GTEx database to examine eQTL of signiicant variants and suggestive variants in each locus. Unfortunately, no eQTL was found in the lead significant variants (Table S3). Then we extended the candidates for including suggestive variants whose *p* value was less than 1.0 × 10^–5^, and also that has strong LD against each significant SNP (*r*^*2*^ > 0.2). There are significant eQTL of *NMNAT1P1* pseudo-gene on TSHR gene locus in rs17111387, rs74771569, rs78176261, which three SNPs had strong LD against rs116622332 (the lead variant in Chromosome 14). Though *NMNAT1P1* itself is a pseudo-gene, many significant eQTL variants are also shared with TSHR and *NMNAT1P1.* Additionally, about the genes around every lead variant, we can find the consistency between the peak of Manhattan plot and the peak of eQTL information about *TSHR* in thyroid (Fig. 3). That means there is a probability that rs116622332 allele on chromosome 14, affects *TSHR* and *NMNAT1P1* expression in thyroid. eQTL analysis identified that rs77317344 ,which is in strong LD with rs14237900 (the lead variant in Chromosome 13), affects the expression of *COG3*. We cannot find other significant information in eQTL analysis about the other genes.

There are some limitations to our study. First, in this study, replication is lacking. Replication studies in other Japanese cohorts and/or other populations are required. Second, many of the individuals analyzed in our study were affected by the Great East Japan Earthquake of 2011. We should consider mental disturbance and stress caused by this big disaster as a confounding factor. To conclude, we investigated the UAE associated SNPs in the Japanese population after adjusting for age, gender, hypertension, and impaired glucose tolerance. The 18 identified SNPs were uncovered to show a statistically significant effect on the UAE. There are limited studies evaluating the association with other candidate genes that we detected. The functional and biological roles exerted by each of the SNPs/genes are required to be elucidated in further studies.

## Electronic supplementary material

Below is the link to the electronic supplementary material.

Supplementary file1 (DOCX 15329 kb)

## Data Availability

The datasets analyzed in this study are not open to the public for ethical reasons but are available upon request after approval of the Ethical Committee of Tohoku University, the Ethical Committee of Iwate Medical University, and the Materials and Information Distribution Review Committee of the TMM Project.
