# Correction to: Genome-wide association study identifies new loci for albuminuria in the Japanese population

**DOI:** 10.1007/s10157-021-02053-4

**Published:** 2021-03-27

**Authors:** Hiroshi Okuda, Koji Okamoto, Michiaki Abe, Kota Ishizawa, Satoshi Makino, Osamu Tanabe, Junichi Sugawara, Atsushi Hozawa, Kozo Tanno, Makoto Sasaki, Gen Tamiya, Masayuki Yamamoto, Sadayoshi Ito, Tadashi Ishii

**Affiliations:** 1 Department of Education and Support for Regional Medicine, Tohoku University Hospital, 1-1 Seiryo-machi, Aoba-ku, Sendai, Miyagi 980-8574 Japan; 2 Tohoku Medical Megabank Organization, Tohoku University, 2-1 Seiryo-machi, Aoba-ku, Sendai, Miyagi 980-8573 Japan; 3 Department of Nephrology, Endocrinology and Vascular Medicine, Graduate School of Medicine, Tohoku University, 1-1 Seiryo-machi, Aoba-ku, Sendai, Miyagi 980-8574 Japan; 4 Radiation Effects Research Foundation, 5-2 Hijiyama Park, Minami-ku, Hiroshima, Hiroshima, 732-0815 Japan; 5 Iwate Tohoku Medical Megabank Organization, Iwate Medical University, 1-1-1 Idaidori, Yahaba-cho, Shiwa-gun, Iwate, 028-3694 Japan; 6 RIKEN Center for Advanced Intelligence Project Nihonbashi, 1-chome Mitsui Bldg. 15F, 1-4-1 Nihonbashi, Chuo-ku, Tokyo, 103-0027 Japan

## Correction to: Clinical and Experimental Nephrology (2020) 24:696–704 10.1007/s10157-020-01884-x

The article “Genome‑wide association study identifies new loci for albuminuria in the Japanese population”, written by Hiroshi Okuda · Koji Okamoto · Michiaki Abe · Kota Ishizawa · Satoshi Makino · Osamu Tanabe · Junichi Sugawara · Atsushi Hozawa · Kozo Tanno · Makoto Sasaki · Gen Tamiya · Masayuki Yamamoto · Sadayoshi Ito and Tadashi Ishii was originally published Online First without Open Access. After publication in volume 24, issue 8, pages 696–704 the author decided to opt for Open Choice and to make the article an Open Access publication. Therefore, the copyright of the article has been changed to © The Author(s) 2021 and the article is forthwith distributed under the terms of the Creative Commons Attribution 4.0 International License (https://creativecommons.org/licenses/by/4.0/), which permits use, sharing, adaptation, distribution and reproduction in any medium or format, as long as you give appropriate credit to the original author(s) and the source, provide a link to the Creative Commons licence, and indicate if changes were made. The original article has been corrected.

