# Supplementary material for: Genome-wide association study identifies new loci for albuminuria in the Japanese population
Source: Clin Exp Nephrol. 2020 Jul 20;24(8):1–9. doi: 10.1007/s10157-020-01884-x (PMC7994224; doi:10.1007/s10157-020-01884-x)
Supplement: Supplementary file 1 — Supplementary file1 (DOCX 15329 kb) [file 10157_2020_1884_MOESM1_ESM.docx]

Supplementary Information for ‘Genome-wide association study identifies New Loci for albuminuria in the Japanese population’

Hiroshi Okuda^1,2,3^, Koji Okamoto^2,3*^, Michiaki Abe^1,2,3^, Kota Ishizawa^1,2^, Satoshi Makino^2^, Osamu Tanabe^2,4^, Junichi Sugawara^2^, Atsushi Hozawa^2^, Kozo Tanno^5^, Makoto Sasaki^5^, Gen Tamiya^2^, Masayuki Yamamoto^2^, Sadayoshi Ito^2,3^, Tadashi Ishii^1,2^.

^1^ Department of Education and Support for Regional Medicine, Tohoku University Hospital, Sendai, Miyagi, Japan

1-1 Seiryo-machi, Aoba-ku, Sendai, Miyagi, 980-8574, Japan

^2^ Tohoku Medical Megabank Organization, Tohoku University, Sendai, Miyagi, Japan

2-1 Seiryo-machi, Aoba-ku, Sendai, Miyagi, 980-8573, Japan

^3^ Department of Nephrology, Endocrinology and Vascular Medicine, Graduate School of Medicine, Tohoku University, Sendai, Miyagi, Japan

1-1 Seiryo-machi, Aoba-ku, Sendai, Miyagi, 980-8574, Japan

^4^ Radiation Effects Research Foundation, Hiroshima-city, Hiroshima, Japan

5-2 Hijiyama Park, Minami-ku, Hiroshima, Hiroshima, 732-0815, Japan

^5^ Iwate Tohoku Medical Megabank Organization, Iwate Medical University, Shiwa, Iwate, Japan

2-1-1 Nishitokuta, Yahaba-cho, Shiwa-gun, Iwate, 028-3694, Japan

^*^Corresponding author

E-mail: okamoto5-tky@umin.ac.jp

Tel: ＋81-22-717-7163

Fax: ＋81-22-717-7163

Supplementary Figures S1 (a) - (m)

Supplementary Tables S1 - S3

**Supplementary Figure Legend**

**Fig S1(a) - (m) Association Signals Around the Significant Loci.**

The upper panel is association signals around the significant loci.

The x-axis represents chromosomal positions (GRC37/hg19) and the y-axis represents −log10 p-values.

The lead variant is shown in purple. Colors represent the degree of LD (r^2^) between each variant and the lead variant.

The LD (r^2^) was calculated based on the combined dataset of TMM subjects.

The lower panels represent the Single - tissue eQTL analyses, where the target was mostly expressed. Data were from GTEx (V8).

The x-axis is represents chromosomal positions (GRC38/hg38) and the y-axis represents −log10 eQTL p-values.

The x-axis between the upper and the lower panel is adjusted by calculating with hgLiftOver [https://genome.ucsc.edu/cgi-bin/hgLiftOver].

(a)*GRM7* locus, (b)*LPA* locus, (c)*PRKAG2* locus, (d) *TPT1-AS1* locus, (e)*EXOC1/NMU* locus, (f)*STEAP1B*/*RAPGEF5* locus, (g)*SEMA3D* locus, (h)*TRIQK* locus, (i)*SERTM1* locus, (j)*OR5AU1* locus, (k)*FMN1*/*RYR3* locus, (l)*COPRS* locus, (m)*BRD1* locus.

**Table S1. The correlation factor of each covariant against urinary albumin excretion.**

|  | CF |
| --- | --- |
| Age | 0.059 |
| BMI | 0.066 |
| SBP | 0.094 |
| UNa | 0.012 |
| UK | 0.027 |
| eGFRcys | 0.11 |
| HbA1c | 0.11 |

[CF: the absolute value of correlation factor against urinary albumin excretion, SBP: systolic blood pressure, UNa: urinary sodium, UK: urinary potassium, eGFRcys: estimated glomerular filtration rate calculated by serum cystatin C, HbA1c (NGSP): hemoglobin A1c valued as National Glycohemoglobin Standardization Program.**]**

**Table S2. The correlation factor between each covariant.**

|  | Age | BMI | SBP | UNa | UK | eGFRcys | HbA1c |
| --- | --- | --- | --- | --- | --- | --- | --- |
| Age |  | 0.096 | 0.26 | 0.069 | 0.0023 | *0.56 | 0.25 |
| BMI | 0.096 |  | *0.48 | 0.072 | 0.020 | 0.17 | 0.19 |
| SBP | 0.260 | *0.48 |  | 0.0056 | 0.080 | 0.14 | 0.12 |
| UNa | 0.069 | 0.072 | 0.0056 |  | *0.44 | 0.093 | 0.020 |
| UK | 0.0023 | 0.019 | 0.080 | *0.44 |  | 0.008 | 0.028 |
| eGFRcys | *0.56 | 0.17 | 0.14 | 0.093 | 0.0080 |  | 0.10 |
| HbA1c | 0.25 | 0.19 | 0.12 | 0.02 | 0.028 | 0.10 |  |

The absolute value of correlation factor between each covariant factor is described in Table S2.

[SBP: systolic blood pressure, UNa: urinary sodium, UK: urinary potassium, eGFRcys: estimated glomerular filtration rate calculated by serum cystatin C, HbA1c (NGSP): hemoglobin A1c valued as National Glycohemoglobin Standardization Program.

*: significant correlation (The absolute value of correlation factor > 0.4)**]**

**Table S3. Significant/Suggestive polymorphisms which are significant in eQTL database.**

| Chr | Significant SNP | Suggestive SNP (r^2^ > 0.2) | Gene | p-value from GTEx (Organ) |
| --- | --- | --- | --- | --- |
| 14 | rs116622332 |  |  |  |
|  |  | rs17111387 | *NMNAT1P1* | 1.10E-11(Thyroid) |
|  |  | rs74771569 | *NMNAT1P1* | 3.20E-12(Thyroid) |
|  |  | rs78176261 | *NMNAT1P1* | 3.20E-12(Thyroid) |
| 6 | rs146871152 |  |  |  |
|  |  | chr6:161096892_C_T |  |  |
|  |  | rs371445781 |  |  |
|  |  | chr6:161032330_G_T |  |  |
|  |  | rs117174672 |  |  |
|  |  | chr6:160815453_G_C |  |  |
| 13 | rs14237900 |  |  |  |
|  |  | rs151183316 |  |  |
|  |  | rs77317344 | *COG3* | 1.90E-5(Breast) |
|  |  | chr13:45776161_C_T |  |  |
| 7 | rs140221313 |  |  |  |
|  |  | chr7:84215995_A_G |  |  |
|  |  | chr7:84327391_G_A |  |  |
|  |  | rs146896786 |  |  |
|  |  | rs78934110 |  |  |
|  |  | chr7:84429171_C_A |  |  |
|  |  | chr7:84506152_G_A |  |  |
|  |  | chr7:84276673_G_T |  |  |

[Chr: Chromosome, Significant SNP: The lead variant in each locus, Suggestive SNP: Significant variant for UAE (p < 1.0 x 10^-5^), which has strong linkage disequilibrium (LD) against each significant SNP (r^2^ > 0.2).]

Genes were selected as significant eQTL by polymorphisms. The data is obtained from GTEx. Blank data mean a lack of information in GTEx.
